# Microbiota-dependent metabolites – New engine for T cell warriors

**DOI:** 10.1080/19490976.2025.2523815

**Published:** 2025-06-30

**Authors:** Yang Tang, Anbo Fu, Liangjing Wang, Qiwei Ge

**Affiliations:** a Department of Gastroenterology, Second Affiliated Hospital of Zhejiang University School of Medicine, Hangzhou, Zhejiang, China; b Institute of Gastroenterology, Zhejiang University, Hangzhou, Zhejiang, China; c Prevention and Treatment Research Center for Senescent Disease, Zhejiang University School of Medicine, Hangzhou, Zhejiang, China

**Keywords:** Gut microbiota, microbiota-dependent metabolites, T cells, molecular mechanisms, clinical translation

## Abstract

Microbiota-dependent metabolites (MDMs) are small bioactive molecules produced or modified through microbial metabolic processes, playing an essential role in the communication between prokaryotic and eukaryotic cells. One of their most important roles is their regulatory effects on the immune system, particularly in shaping the development, differentiation, and function of T cells, which are key players in the adaptive immune response. Emerging research highlights those microbial metabolites, such as short-chain fatty acids (SCFAs), tryptophan-derived metabolites, and bile acids (BAs), modulate T cell responses in both health and disease contexts, impacting conditions ranging from autoimmune disorders to cancer. This review summarizes current advances in deciphering MDMs that critically regulate T cell function and elucidating their biosynthetic origins and mechanisms underlying immunomodulation and pathogenesis. Furthermore, we highlight the application of emerging technologies—*in vitro* bioreactors and organ models, genetic manipulation, and chemical proteomics – in delineating dynamic crosstalk between MDMs and immune signaling networks. We discuss future research perspectives in this field, emphasizing the need for more in-depth mechanistic studies and research strategies from an ecological approach will facilitate the clinical translation of MDMs.

## Introduction

The role of metabolites in biomedical research is rapidly expanding, offering valuable insights for clinical therapies. Notably, immune cells, which play crucial roles in maintaining health and preventing diseases, should not be overlooked. In recent years, clinical therapies targeting T cells have rapidly evolved and been widely adopted. Emerging evidence suggests that T cell activation depends largely on the availability of metabolites. These metabolites are fundamental mediators at each stage of T cell activation, providing distinct signals to regulate these processes. The functional bias of certain metabolites dictates the mode of T cell activation and exhaustion.

Microbiota-dependent metabolites (MDMs) are metabolic byproducts produced by the host-associated microbiota through the processing of dietary compounds, drugs, or other substances ingested by the host. These metabolites not only play a critical role in maintaining the ecological balance of the microbiome but also exert significant effects on the host’s physiology by modulating metabolic, immune, and neurological functions. As central members in the adaptive immune response, T cells are profoundly influenced by MDMs. These influences extend to enhancing CD8^+^ T cell cytotoxicity, modulating cell stemness, maintaining T cell homeostasis, and altering T cell memory potential, which may offer significant therapeutic potential across a wide range of diseases, including cancer, immune-mediated inflammatory diseases (IMIDs) and infections.

Advances in technology have led to the discovery of an increasing number of MDMs and their mechanisms, adding complexity to the understanding of microbiota–host interactions. This review summarizes current knowledge of the interactions between MDMs and T cells, emphasizing the potential of these metabolites as novel regulators of T cell responses. In the near future, MDMs may play a significant role in maintaining tissue homeostasis and treating diseases.

## Microbiota-dependent metabolites

### Short-chain fatty acids

**Figure 1.**
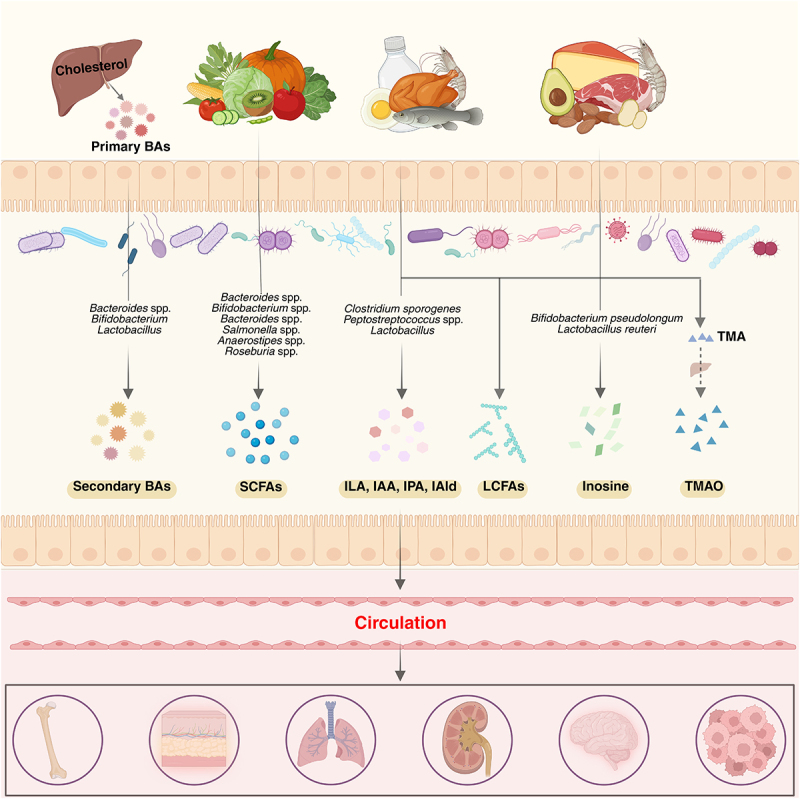
Production of microbiota-dependent metabolites. Gut microbiota-dependent metabolites are derived in three primary ways: from the metabolism of dietary components by gut bacteria, through host-produced compounds that are subsequently modified by the microbiota, or *via de novo* synthesis by gut bacteria. These metabolites can enter systemic circulation, reach various tissues and organs, and exert diverse regulatory effects throughout the body.

Abbreviations: BAs, bile acids; SCFAs, short-chain fatty acids; ILA, indolelactic acid; IAA, indole-3-acetic acid; IPA, indole-3-propionic acid; IAld, indolealdehyde; LCFAs, long-chain fatty acids; TMA, trimethylamine; TMAO, trimethylamine-N-oxide. (Created with bioRender.com)

Short-chain fatty acids (SCFAs) are fatty acids (FAs) with 1 to 6 carbon atoms, primarily produced from the fermentation of undigested dietary fibers by microorganisms in the colon and cecum.^1,2^ The most abundant SCFAs in the intestine are acetate, propionate, and butyrate^3,4^ (Figure 1). Early research on human cases of sudden death has shown that total SCFA concentration (mmol/kg) is relatively low in the terminal ileum (13 ± 6), while it is significantly higher across all regions of the colon, ranging from 131 ± 9 in the cecum to 80 ± 11 in the descending colon.^4^ Acetate is produced from pyruvate *via* acetyl-CoA or the Wood-Ljungdahl pathway by various intestinal bacteria (e.g., *Akkermansia muciniphila*, *Bacteroides* spp., *Bifidobacterium* spp.).^5,6^ Propionate is synthesized through three pathways – succinate, acrylate, and propanediol – by specific microorganisms like *Bacteroides* spp. and *Salmonella* spp.^2,7^ Butyrate is formed from two acetyl-CoA molecules through two enzymatic routes involving bacteria such as *Anaerostipes* spp. and *Roseburia* spp.^2^ (Figure 1).

Following their production, SCFAs are absorbed *via* passive diffusion or carrier-mediated transport (mainly MCT1 and SMCT1, more SCFA transport receptors are reviewed by Boushra et al.) into the bloodstream.^8,9^ However, due to significant liver metabolism, only a small fraction of SCFAs reaches systemic circulation and peripheral tissues.^4,10,11^ Plasma concentrations of acetate, propionate, and butyrate in human peripheral venous blood are estimated to be 19–146 μM, 1–13 μM, and 1–12 μM, respectively.^4^

SCFAs can enter cells directly to serve as energy substrates or inhibit histone deacetylases (HDACs).^2,12^ Additionally, SCFAs can bind to G protein – coupled receptors (GPCRs), such as G-protein-receptor (GPR) 41, GPR43, and GPR109A, thereby modulating signal transduction.^13–15^ Early research has established that SCFA-sensing GPCRs are predominantly expressed in innate immune populations – including macrophages, dendritic cells (DCs), and intestinal regulatory T cells (Tregs) – but are largely absent in most conventional T lymphocytes.^2,15^ This expression pattern leads to the prevailing hypothesis that SCFAs modulate adaptive immunity indirectly through innate intermediaries.^16–18^ Strikingly, subsequent studies challenge this paradigm by demonstrating functional GPCR expression on differentiated effector T cells, revealing direct SCFA-GPCR signaling crosstalk in these populations.^19–21^

Through these mechanisms above, SCFAs influence various cellular functions including energy metabolism^22–24^ and immune responses.^14,25–27^

### Tryptophan and indole derivatives

Tryptophan is an essential amino acid obtained from dietary protein. While most proteins are digested and then absorbed in the small intestine, some proteins and amino acids reach the colon,^28^ where they are metabolized by the host and gut microbiota into various metabolites (Figure 1).

In the human body, tryptophan is metabolized *via* three main pathways: the kynurenine (Kyn) pathway, the serotonin (5-hydroxytryptamine) pathway, and the indole pathway.^29^ Microorganisms primarily metabolize tryptophan through the indole pathway, producing various indole derivatives.^29^ *Clostridium sporogenes* is reported to be associated with the production of indole pyruvic acid, indole-3-lactic acid (ILA), and indole-3-propionic acid (IPA).^30,31^ The fldC subunit, an important phenyllactate dehydratase subunit in *C. sporogenes*, is indispensable for IPA biosynthesis.^30^ The reductive metabolism of aromatic amino acids proceeds through a conserved pathway mediated by the homologues of phenyllactate dehydratase in these microorganisms.^30^ *Peptostreptococcus* spp. including *P. russellii, P. anaerobius*, and *P. stomatis*, could convert tryptophan to indoleacrylic acid (IA) and IPA.^32^ *Lactobacillus* species are also significant sources of microbial tryptophan metabolites, converting tryptophan to indolealdehyde (IAld) and ILA.^33,34^ Additionally, some microorganisms can produce kynurenine (Kyn), serotonin (5-Hydroxytryptamine, 5-HT) and their downstream metabolites, such as 3-hydroxyanthranilic acid (3-HAA) and 3-hydroxykynurenine (3 H-Kyn)^35^ (Figure 1).

The average serum concentrations of microbial indole derivatives are estimated to be 60–80 µM (IPA and indolepyruvic acid) and 0–20 µM (IAA and ILA) in mice.^30^ In human, mean concentrations in healthy adults have previously reported for IAA (227 ng/ml), IPA (191.1 ng/ml), and ILA (31.5 ng/ml).^36^ These indole derivatives could act as bioactive compounds, facilitating communication between bacteria and the host mainly by binding to specific receptors like the aryl hydrocarbon receptor (AhR) and pregnane X receptor (PXR).^33,37–39^

### Bile acids

Microbial bile acids (BAs) refer to BAs that are produced by the human body and modified by microorganisms.^40^ BAs are synthesized by hepatocytes through cholesterol oxidation.^41^ In human, BAs can be classified into primary and secondary types, both of which aid in nutrient digestion, transport, and absorption. Primary BAs, such as cholate (CA) and chenodeoxycholate (CDCA) (hydrophobic), are synthesized in the liver, conjugated with glycine or taurine (hydrophilic), and subsequently secreted into the intestinal lumen.^41,42^ About 95% of these BAs are reabsorbed in the terminal ileum and return to the liver *via* enterohepatic circulation, while 5% are metabolized by gut microbiota in the cecum and colon into secondary BAs^43^ (Figure 1).

Microbial transformation of BAs involves four key reactions: deconjugation, dehydroxylation, oxidation, and epimerization. Deconjugation, the initial and crucial step, is mediated by bile salt hydrolases (BSHs), enzymes found in many bacteria, including *Bacteroides* spp., *Bifidobacterium*, and *Lactobacillus*.^44,45^ Dehydroxylation, primarily carried out by anaerobic bacteria such as *Clostridium*,^46^ is essential for converting primary BAs into secondary BAs like deoxycholic acid (DCA) and lithocholic acid (LCA). The oxidation of BAs to oxo-BAs is a key step preceding epimerization. These two processes are mediated by different hydroxysteroid dehydrogenases (HSDHs) in various microorganisms, such as 3α/β-HSDH, 7α/β-HSDH, and 12α/β-HSDH,^44,47^ which contribute to the diversification of microbial BAs. Generally, oxidation and epimerization require the coordinated effort of two HSDHs: hydroxyl groups are oxidized by position-specific HSDHs (e.g., 7α-HSDH), and then reduced by another position-specific HSDH, 7β-HSDH.^48^ These two reactions can be performed by the same or different microorganisms.^48^ Intriguingly, recent research has identified the fifth microbial modification to produce re-conjugated BAs.^49^ These re-conjugations are independent of glycine or taurine, producing compounds such as phenylalanocholic acid, tyrosocholic acid, and other BA amidates^50,51^ (Figure 1).

BAs and their derivatives engage with specific receptors, including the farnesoid X receptor (FXR), PXR, vitamin D receptor (VDR), and G protein-coupled bile acid receptor 1 (GPBAR1).^42^

### Others

Beyond modulating SCFAs, microorganisms also influence long-chain fatty acids (LCFAs). Essential LCFAs for human, such as linoleic acid (LA) and linolenic acid (LNA), are primarily derived from dietary intake. Commensal microorganisms metabolize these FAs into a series of microbial FA isomers, including conjugated LAs (CLAs) and conjugated LNAs (CLNAs).^52^ The process is mainly mediated by linoleate isomerase (LAI) encoded by gut microbiota^52^ (Figure 1).

Another important MDM is trimethylamine (TMA), produced from the metabolism of dietary choline and L-carnitine found in red meat and fish (Figure 1). TMA is absorbed by the host and converted into trimethylamine N-oxide (TMAO) *via* hepatic flavin monooxygenases.^53,54^ Additionally, gut microbiota is involved in the metabolism of vitamins.^55^ Recent studies have also shown that gut microbiota increase the abundance of plasma adenosine metabolites, such as inosine.^56^ The elevation of inosine may result from increased production by *Bifidobacterium pseudolongum*^57^ or enhanced intestinal absorption and transport by *Lactobacillus reuteri*.^58^

## Microbiota-dependent metabolites in T cell regulation

**Figure 2.**
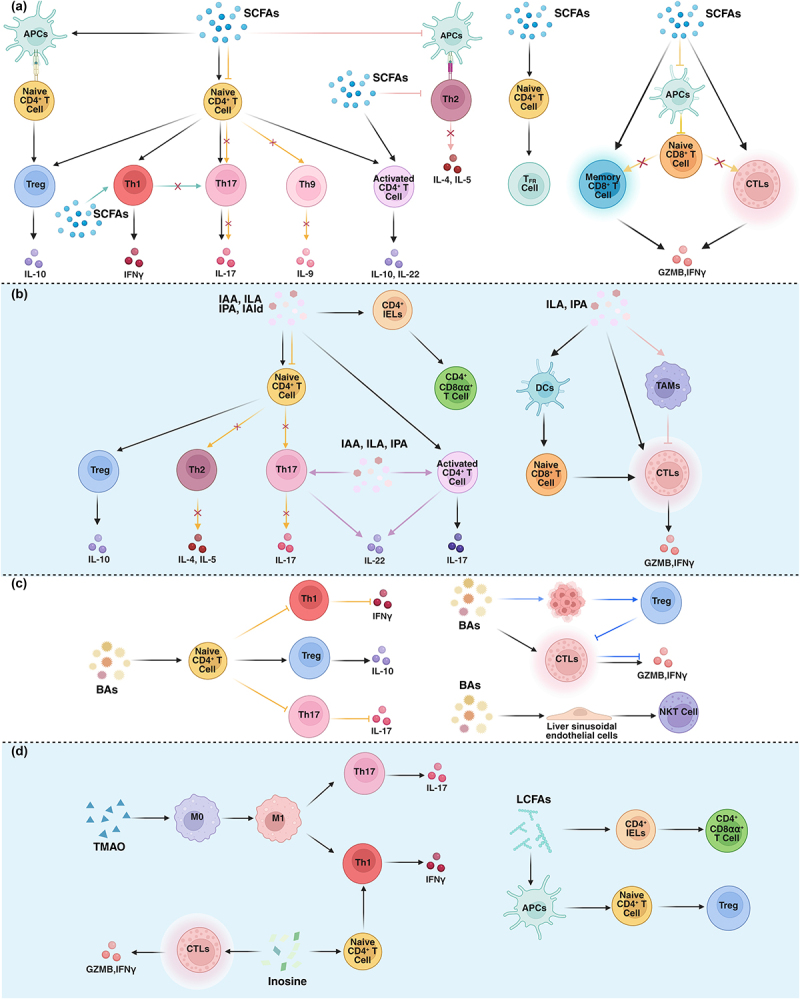
Effects of microbiota-dependent metabolites on T cells. Microbiota-dependent metabolites modulate T cell functions in diverse ways, influencing CD4^+^ T cells, CD8^+^ T cells, NKT cells, and their respective subtypes. The arrows in the same color represent a continuous regulatory process.

Abbreviations: APCs, antigen-presenting cells; BAs, bile acids; CTLs, cytotoxic T lymphocytes; GZMB, granzyme B; IAA, indole-3-acetic acid; IAld, indolealdehyde; IELs, intraepithelial lymphocytes; IFNγ, interferon-γ; IL, interleukin; ILA, indolelactic acid; IPA, indole-3-propionic acid; LCFAs, long-chain fatty acids; M0, naïve macrophages; M1, classical-activated macrophages; NKT cells, natural killer T cells; SCFAs, short-chain fatty acids; TAMs, tumor-associated macrophages; T_FR_ cells, follicular regulatory T cells; Th, T helper cells; TMAO, trimethylamine-N-oxide; Treg, regulatory T cell. (Created with bioRender.com)

T cells are central to adaptive immunity, orchestrating cell-mediated immune responses to maintain host health and prevent diseases. T cell development primarily occurs in the thymus, where T cells differentiate into various subtypes, including CD4^+^ and CD8^+^ αβ T cells, γδ T cells, natural killer T (NKT) cells, and thymus regulatory T cells (tTregs).^59^ In addition to cells differentiated from the thymus, cytokines or peripheral stimuli can also induce other cell subtypes, such as peripheral induced Tregs (pTregs) and T helper 17 cells (Th17).^59^ T cells are known to be intricately regulated by various metabolites that support the distinct requirements of each differentiation state, lineage, and function.^60,61^ Recent studies have further suggested that MDMs can modulate T cell function through receptor binding or direct entry into cells.^31,62^ Here, we integrate emerging insights into MDMs, highlighting their pleiotropic effects on divergent T cell subsets (Figure 2).

### SCFAs

SCFAs have become a prominent focus for their effects on the immune system.^2,15,26,63^ Here, we will focus on their roles in enhancing the differentiation and function of Tregs, as well as their involvements in controlling Th17, other CD4^+^ T cells, and CD8^+^ T cells (Figure 2a). Herein, we summarize the immunomodulatory mechanisms of SCFAs including: 1) epigenetic regulation through histone deacetylase (HDAC) inhibition, which modulates chromatin accessibility and transcriptional activity; and 2) receptor-mediated signaling *via* activation of G-protein-coupled receptors (GPCRs) including GPR41, GPR43, and GPR109A, triggering downstream signaling cascades that mediate cellular responses.

#### Th17/Tregs

Th17 and Tregs represent two distinct phenotypes of CD4^+^ T cells with completely different functions: pro-inflammatory (Th17) and anti-inflammatory (Tregs). The balance between these two subsets is crucial for maintaining immune homeostasis.^64^

SCFAs have been shown to induce the extrathymic differentiation of Tregs, also known as pTregs, *via* GPCR activation and HDAC inhibition.^16,25,62,65,66^ Acetate promotes pTreg by inhibiting HDAC9 to enhance acetylation at Foxp3.^67^ Butyrate treatment is suggested to promote colonic Treg differentiation and mitigates colitis by enhancing histone H3 acetylation at the Foxp3 locus, and the process is independent of GPR43.^65^ In fact, the effect of butyrate on Treg differentiation is context-dependent, requiring the presence of transforming growth factor-β (TGF-β). In the absence of TGF-β, butyrate fails to promote Foxp3 expression and Treg differentiation, and at higher concentrations, it even promotes Th1 differentiation by enhancing acetylation at the Ifng and Tbx21 loci.^68^ It is worth mentioning that, contrary to the conclusion regarding the absence of GPR43 on Tregs made by Furusawa et al., Smith et al. suggest that SCFAs especially propionate promotes colonic pTreg *via* GPR43.^62^

SCFAs also indirectly influence Tregs by modulating other immune cells, such as antigen-presenting cells (APCs). For example, propionate acts on CD11c^+^ dendritic cells (DCs), endowing them with regulatory functions that promote the conversion of naive CD4^+^ T cells into pTregs.^16^ Activation of GPR109A on colonic macrophages and DCs by butyrate further induces anti-inflammatory responses, increasing Treg and interleukin (IL) −10-producing CD4^+^ T cell frequencies in the colon, thereby reducing colitis.^17^

Regarding Th17, interestingly, research has found that SCFA-induced Foxp3^+^ Treg development occurs only under condition of low T cell activation (anti-CD3 1 μg/mL), but not under Th1 or Th17 polarization condition characterized by high anti-CD3 activation levels,^66^ supporting that SCFAs play a role in regulating the Th17/Treg balance. Conversely, while SCFAs can enhance Th17 generation during active immune responses,^66,69,70^ they can also inhibit Th17 cell proliferation and development by suppressing key mediators including IL-17A, retinoic acid receptor-related orphan nuclear receptors gamma t (RORγt), and other Th17-related factors, leading to metabolic and epigenetic reprogramming that diminishes the pathogenic phenotype of Th17.^71,72^ SCFAs can also prevent the conversion of Th1 cells to Th17 cells by enhancing T-bet expression.^68^ These findings indicate that SCFAs modulate the Th17/Treg balance in a context-dependent manner.

#### Other CD4^+^ T cells

IL-10 is a key immunosuppressive cytokine produced by Tregs and other CD4^+^ T cells, serving as a self-regulatory mechanism to prevent excessive T-cell responses. Some research shows that SCFAs regulate the differentiation of naive CD4^+^ T cells into IL-10-expressing T cells through HDAC inhibition, rather than *via* GPR43/GPR41 pathways.^66,71^ This process involves the mTOR-S6K pathway and the transcription factor Blimp-1, which govern the expression of key effector cytokines like IL-10, interferon-γ (IFN-γ), and IL-17.^66,71^ In contrast, another group has shown that butyrate activates STAT3 and mTOR, upregulating Blimp-1 to induce IL-10 production in differentiated Th1 effector cells *via* a GPR43-dependent mechanism.^21^ The expression of GPR43 is very low in naïve T cells but relatively higher in differentiated Th1 effector cells, which may account for the different mechanisms of butyrate on differentiated Th1 effector cells and naïve T cells.^71^ SCFAs also promote IL-22 production in CD4^+^ T cells *via* both GPR41 activation and HDAC inhibition, enhancing the expression of hypoxia inducible factor 1 alpha (HIF-1α) and aryl hydrocarbon receptor (AhR), which are crucial for IL-22 regulation.^27^

SCFAs also broadly regulate other CD4^+^ T cells. For instance, propionate reduces pro-inflammatory cytokine secretion by effector CD4^+^ T cells, such as IL-4, IL-5, and TNF-α, in a mouse dermatitis model.^16^ Butyrate decreases IL-4 production in Th2-polarized T cells, thereby alleviating allergic lung inflammation in a mouse model.^73^ Propionate also reduces susceptibility to allergic airway inflammation by enhancing the function of bone marrow-derived DCs, impairing their ability to activate Th2 effector cells in the lung.^18^ The process is mediated by GPR41.^18^ Additionally, butyrate has been found to decrease Th9 cell frequency in the lung, reducing lung inflammation.^74^ Recent research highlights that SCFAs may also directly influence T cell metabolism. The reduction of SCFAs impairs mTOR activity and mitochondrial function in mucosal CD4^+^ T cells, leading to T cell dysfunction and metabolic adaptations.^75^

#### CD8^+^ T cells

SCFAs regulate CD8^+^ T cell metabolism. SCFAs can be transported into cells *via* MCT1 or SMCT1 receptors, or through passive diffusion.^8,9^ Through these processes, SCFAs can directly serve as substrates for ATP production or indirectly promotes cytoplasmic and nuclear protein acetylation, which critically enhance metabolism and function in immune cells.^10^ Recent studies have shown that elevated systemic acetate level following acute bacterial infection in mice is essential for optimal memory CD8^+^ T cell function, particularly IFN-γ production.^76^ Mechanically, acetate absorption by memory CD8^+^ T cells expands the cellular acetyl-coenzyme A pool *via* ATP citrate lyase, promoting GAPDH acetylation and enhancing glycolysis, thereby boosting rapid memory CD8^+^ T cell responses.^76^ Similar effects of SCFAs in enhancing IFN-γ production as metabolic substrates in cytotoxic T lymphocytes (CTLs) have been confirmed in other studies.^77,78^ A study performed on mice have found that feeding a high-fiber diet could increase mitochondrial mass and glucose transporter 1 (GLUT1) surface expression in CD8^+^ T cells during influenza infection, leading to enhanced glycation rates and oxidative phosphorylation (OXPHOS) activity, ultimately strengthening CD8^+^ T cell responses.^78^ These metabolic changes are partially mediated by SCFAs through GPR41 and GPR43. Notably, GPR41 and GPR43 are crucial for enhancing multiple functions of CD8^+^ T cells including the killing function, memory potential, and recall capacity upon antigen re-encounter.^19,20^

Moreover, SCFAs can modulate CD8^+^ T cells in ways beyond metabolic regulation. Butyrate inhibits HDAC, which induces the expression of transcription factors like ID2, thereby enhancing IL-12 signaling and promoting CTL cytotoxicity.^77,79^ Pentanoate, a rare SCFA produced by low-abundance commensals like *Megasphaera massiliensis*, also modulates CTL effector molecule expression by inhibiting HDAC and enhancing mTOR complex activity, independent of GPR41/GPR43.^80^

SCFAs also affect CD8^+^ T cell function indirectly by modulating APCs. Supplementing SCFAs can inhibit DC secretion of IL-12, which is crucial for the differentiation of naive CD8^+^ T cells into antigen-specific memory and effector CTLs, thereby limiting antigen-specific CD8^+^ T cell activation.^81^ SCFAs have been shown to promote CD8^+^ T cell proliferation and survival, prevent CD8^+^ T cell exhaustion, and facilitate the conversion of Tc17 cells to the CTL phenotype, independent of GPR41/GPR43 signaling.^77,79,80^

#### Other T cells

Follicular regulatory T (T_FR_) cells, serving as regulators of the germinal center reaction, counteract the functions of follicular helper T (T_FH_) cells. T_FH_/T_FR_ balance is crucial in the maintenance of immune homeostasis. Butyrate directly induces the differentiation of functional T_FR_ cells and further suppresses autoantibody production in the systemic lymphoid tissue.^82^ This effect is attributed to HDAC inhibition by butyrate, leading to histone hyperacetylation in the promoter region of the T_FR_-cell marker genes.^82^

### Tryptophan and indole derivatives

Increasing studies highlight the pivotal role of tryptophan metabolites produced by microorganisms in regulating T lymphocyte immunity. These metabolites help maintain a balance between various T cell immune responses in many ways: through aryl hydrocarbon receptor (AhR), directly participating in cellular activities, and influencing intestinal microecology.^29,35^ AhR, a transcription factor widely expressed in immune cells, has been extensively studied for its critical role in the development and differentiation of T cells^83–85^ (Figure 2b). The diversity of tryptophan metabolites and their varying affinities for AhR may determine the specific induction of different T cell subsets.

#### Th17/Tregs

Dysregulation of microbiota-associated tryptophan metabolism has been implicated in diseases caused by Th17/Treg imbalances.^31,86–89^ Supplementing dietary tryptophan has been shown to increase Foxp3 expression and reduce IL-17 production by restoring microbial AhR ligand production, possibly involving tryptamine.^86^ All these observations implicate the roles of microbiota-associated tryptophan metabolism in Th17 and Treg regulation.

Jiang et al. have shown that sinomenine (SIN), an immunosuppressive agent used in rheumatoid arthritis (RA) treatment, restores intestinal microbial balance, leading to increased production of indoleacrylic acid (IA), indole-3-propionic acid (IPA), and indole-3-acetic acid (IAA).^90^ These metabolites modulate NF-κB and MAPK phosphorylation through AhR activation, thereby decreasing Th17 cells, increasing Tregs, and restoring Th17/Treg balance.^90^ Breast milk-promoted Bifidobacterium species produce aromatic lactic acids in infants’ guts, particularly indole-3-lactic acid (ILA), which is responsible for IL-22 production by Th17 cells after AhR stimulation.^91^ This suggests that microbial metabolites may influence early immune function in infants, consistent with findings from other studies.^92^ IAA also alleviates colitis by promoting IL-22 production in colonic T cells *via* AhR activation in mice.^31^ The conversion of tryptophan to IAA is impaired with CARD9 deficiency, a susceptibility gene for inflammatory bowel disease (IBD),^31^ indicating a complex interplay between host immune-related genes and microbiota metabolism.

*Lactobacillus reuteri* has been reported to increase the frequency of central nervous system infiltrating myelin-reactive CD4^+^ T cells and the production of IFN-γ and IL-17.^93^ Some research has confirmed that *L. reuteri* metabolizes tryptophan into various derivatives, including indoleacetate, indole-3-glyoxylic acid, tryptamine, p-cresol, and diverse imidazole compounds, which enhance IL-17-producing T cells *via* AhR activation, thus contributing to central nervous system autoimmunity.^88^ However, other studies suggest that supplementation with ILA, IAA, and indolealdehyde (IAld) produced by *Lactobacillus murinus* reduces Th17 polarization in a dose-dependent manner, alleviating salt-induced exacerbation of experimental autoimmune encephalomyelitis and salt-sensitive hypertension.^34^ These findings imply that different tryptophan metabolites may exert their effects through different mechanisms in immune cells. The research of Henrick et al. further support these results: ILA does not always activate AhR; it can exert direct immunoregulatory effects by inducing galectin-1 in Th2 and Th17 cells during helper T cell polarization, thereby limiting T cell activation.^92^ In fact, many of ILA’s effects on Th17 cells are not mediated by AhR. For instance, statins increase *L. reuteri* and ILA levels, which further suppress Th17 differentiation by inhibiting the transcriptional activity of RORγt, the master transcription factor of Th17 cells. This inhibition occurs through direct physical interaction with RORγt, reducing its occupancy at targets such as Il-17a and Il-23.^94^

#### Other CD4^+^ T cells

A 2016 study has identified IAld, a tryptophan metabolite derived from skin microbiota, as an activator of AhR that suppresses abnormal Th2-biased immune responses (such as TSLP, IL-4, and IL-5 secretion).^95^ Subsequent research confirms that *Limosilactobacillus reuteri* and *Bifidobacterium longum* regulate gut microbial composition and enhance tryptophan metabolism to produce AhR ligands like ILA, IPA, and indole-3-carbaldehyde (I3C). These ligands activate AhR signaling to reduce aberrant Th2 responses and may offer an effective strategy for alleviating atopic dermatitis (AD).^96,97^ Beyond serving as AhR activators, tryptophan metabolites also directly influence T cell metabolism; for instance, microbial-derived tryptamine promotes mTOR activation and glycolysis in CD4^**+**^ T cells, leading to an enhanced inflammatory phenotype.^98^

#### CD8^+^ T cells

The regulation of CD8^**+**^ T cell function by microbial tryptophan metabolites has garnered increasing attention. These metabolites may influence CD8^+^ T cells by mediating epigenetic modifications.^99,100^ For instance, ILA produced by *Lactobacillus plantarum* L168 enhances the function of tumor-infiltrating CD8^**+**^ T cells by transcriptionally inhibiting Saa3 expression, which is related to cholesterol metabolism, through reduced chromatin accessibility.^99^ IPA modulates the stemness program of CD8^**+**^ T cells and promotes the generation of progenitor exhausted CD8^**+**^ T cells (Tpex) by increasing H3K27 acetylation at the super-enhancer region of Tcf7.^100^

Tryptophan derivatives can also indirectly regulate CD8^+^ T cell function by affecting other immune cells. ILA can enhance IL-12a production in dendritic cells (DCs) by increasing H3K27ac binding at the enhancer regions of IL-12a, thereby contributing to the priming of CD8^+^ T cell immunity against tumor growth.^99^ However, gut-produced ILA and IAA, which accumulate in the tumor microenvironment (TME), can drive an immunosuppressive phenotype in tumor-associated macrophages (TAMs) and suppress CD8^+^ T cell accumulation in the TME *via* AhR stimulation.^38^

#### Other T cells

A unique subset of CD4^+^ T cells, known as double-positive intraepithelial lymphocytes (DP IELs), originates from the lamina propria in the small intestine epithelium. These CD4^+^ CD8αα^+^ TCRαβ T cells have regulatory functions complementary to those of Tregs and promote tolerance to dietary antigens. *Lactobacillus reuteri* provides indole derivatives of dietary tryptophan, such as ILA, which activates AhR, leading to downregulation of the transcription factor Thpok and reprogramming of CD4^+^ IELs into CD4^+^CD8αα^+^ T cells.^33^

### Bile acids

BAs serve as a regulator in T lymphocyte immunity, with recent research focusing on microbial BAs^40,101^ (Figure 2c). BAs exert their immune regulatory effects through various BA receptors of T cells, including FXR, PXR, VDR, RORγt, and GPBAR1.^40,42^

#### Th17/Tregs

Microbial BAs, including 3-oxoLCA, isolithocholic acid (isoLCA), 3β-hydroxydeoxycholic acid (isoDCA), and isoallolithocholic acid (isoalloLCA), regulate the differentiation and development of intestinal Th17 and Tregs.

*Bacteroides uniformis* has been shown to reshape the intestinal microbiota and alter the colonic BA profile, which may inhibit Th17 differentiation in colonic epithelial cells and suppress downstream NF-κB and MAPK inflammatory signaling pathways.^102^ *Eubacterium lentum* and *Ruminococcus gnavus* convert LCA to 3-oxoLCA *in vivo* through their 3α/β-HSDHs enzymes.^103^ IsoLCA, an isomer of LCA, and 3-oxoLCA inhibit Th17 cell differentiation by directly binding to RORγt, a key transcription factor, and suppressing its activity.^103,104^

On the other hand, Bacteroidetes species further metabolize 3-oxoLCA into isoalloLCA.^105^ IsoalloLCA enhances mitochondrial reactive oxygen species (mitoROS) production and increases H3K27 acetylation at the Foxp3 promoter region, which boosts the expression of Foxp3, thereby promoting Treg differentiation. This process also requires TGF-β-induced signaling.^104^ IsoalloLCA has been reported to induce conserved noncoding sequence (CNS) 3-regulated histone acetylation in the enhancer region of the Foxp3 gene and increase the nuclear hormone receptor NR4A1 binding to the Foxp3 promoter, explaining the enhanced Foxp3 expression.^104,105^ The effect of isoalloLCA on Foxp3 appears to be independent of BA receptors. Conversely, other research suggests that microbial BA metabolites (LCA/3-oxoLCA) may regulate RORγ^+^ Tregs in a BA receptor-dependent manner, increasing colonic RORγ^+^ Treg cell counts and reducing susceptibility to inflammatory colitis.^106^ Similarly, isoDCA induces pTregs in an FXR-dependent manner by acting on DCs to reduce their immunostimulatory properties and thereby potentiate Treg generation.^107^ Moreover, isoDCA-producing bacterial consortia have been found to increase the number of colonic RORγt-expressing Tregs in a CNS1-dependent manner, suggesting enhanced extrathymic differentiation.^107^ Reverse metabolomics also reveals that bacteria produce novel bile amidates (Met-CDCA, Met-DCA, Phe-CDCA, tryptophan-CDCA, and Tyr-CDCA) that induce IFN-γ in RORγt^+^CD4^+^ T cells, possibly through binding to FXR.^51^

#### Other CD4^+^ T cells

LCA is first discovered to control adaptive immunity by inhibiting Th1 activation. Specifically, LCA inhibits STAT1 and T-BET in CD4^+^ T cells by binding to VDR, thereby suppressing Th1 differentiation and reducing Th1 inflammatory cytokine secretion.^108^ Additionally, LCA and DCA decrease the proportion of Th1 and Th17 cells in the spleen.^109^ Beyond direct regulation of CD4^+^ T cells, early studies have suggested that taurocholic acid, a substrate for bacterial metabolism, might generate new substances (possibly H_2_S or secondary bile acids) that induce Th1-type immune responses by activating DCs in IL10^−/−^ mice.^110^

#### CD8^+^ T cells

A recent study shows that DCA concentration negatively correlates with CD8^+^ T cell function in colorectal cancer (CRC) patients, with DCA suppressing CD8^+^ T cell responses by targeting plasma membrane Ca^2+^ ATPase (PMCA) to inhibit Ca^2+^-NFAT2 signaling.^7^ This effect can be abolished by the ablation of bacterial DCA biosynthetic pathways.^7^ Moreover, microbial BAs can alter tumor cells to regulate CD8^+^ T cell responses in antitumor immunity. BSH-expressing Bacteroides promote the production of DCA and LCA, which activate GPBAR1, leading to increased β-catenin-regulated CCL28 expression in CRC to elevate intra-tumoral immunosuppressive CD25^+^ Foxp3^+^ Tregs and thus decreasing CD8^+^ T cells.^111^

#### Other T cells

Research published in 2018 has suggested synthesized by *Clostridium* spp. *Clostridium* suppress CXCL16 levels on liver sinusoidal endothelial cells, consequently diminishing CXCR6-dependent NKT cell recruitment in the liver.^112^ Additionally, GPBAR1 is present on NKT cells, where it redirects NKT cell polarization from pro-inflammatory IFN-γ-producing type I NKT cells toward anti-inflammatory IL-10-producing type I NKT cells and increases IL-10-producing type II NKT cells in an IL-10-dependent manner.^113^ This suggests that microbial BAs may exert direct control over NKT cell functions.

### Other metabolites

Trimethylamine N-oxide (TMAO) increases mitoROS and promotes NF-κB nuclear translocation, leading to NLRP3 inflammasome activation in macrophages, which polarizes them to the M1 (classical activated macrophages) type. These M1 macrophages further stimulate Th1 and Th17 differentiation.^114^ TMAO also enhances CD8^+^ T cell-mediated antitumor immunity in triple-negative breast cancer (TNBC) by inducing pyroptosis in tumor cells through activating the endoplasmic reticulum stress kinase PERK^115^ (Figure 2d).

Inosine, an adenosine receptor agonist, modulates immunity by inhibiting Th1/Th2 differentiation and exerting anti-inflammatory effects *via* A2A receptors in Treg-deficiency-induced autoimmunity.^116^ Intriguingly, inosine can also induce Th1 differentiation in gut-associated lymphoid tissue in the absence of anti-CTLA-4, while in combination with anti-CTLA-4, it activates IFN-γ-producing Th1 and CD8^+^ effector T cells.^57,117^ The effect of inosine on T cells requires sufficient co-stimulation, IL-12 receptor engagement for Th1 differentiation, and IFN-γ production for efficient antitumor immunity.^57^

Conjugated linoleic acids (CLAs), isomers of linoleic acids, can induce a distinct small intestinal TCRβ^+^CD4^+^ IELs that express CD8αα homodimers, known as CD4^+^CD8αα^+^ IELs, which are related to maintaining gut homeostasis.^118^ Mechanistically, CLAs bind to the transcription factor HNF4G, promoting intrinsic *Il18r1* transcription in CD4^+^CD8αα^+^ IELs. IL-18 signaling downregulates the CD4 lineage-committed transcription factor ThPOK, supporting CD4^+^CD8αα^+^ IEL development.^118^ Additionally, 12,13-diHOME, a terminal product of linoleic acid produced by bacteria *via* epoxide hydrolase, reduces IL-10 secretion by DCs, thereby decreasing Treg amounts^119^ (Figure 2d).

Ascorbate (vitamin C), a microbial metabolite of bacterial origin, suppresses T effector cells (including IL-17A-, IL-4-, and IFN-γ-producing cells) and inhibits T cell activation by selectively targeting glycolytic-dependent activated T cells through GLUT1.^120^ Moreover, bacterial riboflavin (vitamin B2) metabolites can specifically and potently activate mucosal-associated invariant T (MAIT) cells by competitively binding to MHC class I like related molecule (MR1), indicating that these vitamin B metabolites represent a class of antigens presented by MR1 for MAIT cell immunosurveillance.^121^

## Microbiota-dependent metabolites related diseases

### Cancer

While cancers have conventionally been attributed to somatic mutations and environmental factors, emerging evidence now positions MDMs as regulators of tumorigenesis and tumor progression. Immune cells, especially CD8^+^ T cells, play a pivotal role in the development, treatment, and prognosis of cancers.^122^ Emerging research suggests that MDMs can affect tumor progression by regulating T cells, proposing new insights and strategies for the cancer treatment.

Microbial butyrate boosts the antitumor cytotoxic CD8^+^ T cell response^80,123^ and further enhances the response to chemotherapy to suppress tumor progression.^79^ Elevated serum butyrate level in the oxaliplatin responder confirms this effect.^79^ Moreover, pentanoate and butyrate can also promote the anti-tumor activity of chimeric antigen receptor (CAR) T cells,^80^ thus enhancing the adoptive immunotherapy. Apart from the regulation of CD8^+^ T cells, SCFAs also increase colonic Th17 cell frequency and IL-17A and IL-17F expression. IL-17A expression prior to tumor formation and potentiates intestinal tumorigenesis.^70^

The roles of dietary tryptophan in tumor treatment have garnered significant attention, though its effects remain somewhat unclear. On the one hand, indole-3-lactic acid (ILA) could enhance the function of tumor infiltrating CD8^+^ T cells by increasing the secretion of IL-12a by DCs in peripheral blood, thereby inhibiting tumor growth.^99^ Conversely, ILA and indole-3-acetic acid (IAA) have been shown to accumulate in the TME, driving TAM inhibition phenotype, reducing CD8^+^ T cell and promoting pancreatic tumor growth.^38^ The analysis found that low aryl hydrocarbon receptor (AhR) expression may be beneficial for survival.^38^ Prior investigations have established that ILA directly induces apoptosis in colorectal cancer (CRC).^124^ Further, Han et al. demonstrate that atorvastatin could orchestrate the *L. reuteri*–ILA – IL-17 signaling pathway to suppress CRC development, offering a potential complement to chemoprevention strategies.^94^ Our group reported that IPA, produced by a collaboration between *Lactobacillus johnsonii* and *Clostridium sporogenes*, significantly improved immune checkpoint blockade (ICB) responsiveness across various cancer types.^100^ This is the first study to report the continuous metabolism of amino acids in the diet by two commensal bacteria, and their involvement in improving the treatment response of host diseases.

In CRC patients, elevated deoxycholic acid (DCA) levels correlate with impaired CD8^+^ T cell function. Bacteria harboring DCA biosynthetic genes suppresses CD8^+^ T cells effector function and promoted tumor growth in mice.^7^ These microbial BAs, such as lithocholic acid (LCA) and DCA, promote CRC growth by increasing immunosuppressive CD25^+^Foxp3^+^ regulatory T cells within tumors while suppressing anti-tumor CD8^+^ T cell responses.^7,111^ Though previous studies have reported that high concentrations of BAs can lead to cell death,^125^ Cong et al. further confirm that the DCA concentration required to induce cell death does not fully account for the observed effects of DCA-induced suppression of CD8^+^ T cell function.^7^ Abnormal changes in the concentration of microbial BAs have also been found in liver cancer patients.^112^ The depletion of BA-metabolizing microbiota by antibiotic cocktail promotes CXCL16 expression in the liver, which further enhances hepatic NKT cell accumulation and decrease liver tumor growth in mice.^112^

Multi-omics analyses have shown that patients with higher plasma TMAO in triple-negative breast cancer (TNBC) respond better to immunotherapy, with TMAO being more abundant in tumors with an activated immune microenvironment.^115^ Inosine also enhances ICB therapeutic efficacy by promoting IFN-γ-producing Th1/CD8^+^ effector T cells.^57^

### Immune-mediated inflammatory diseases

Immune-mediated inflammatory diseases (IMIDs) are a heterogeneous group of diseases characterized by chronic inflammation and organ damage.^126^ In the past few decades, emerging research has elucidated the role of MDMs in modulating IMID. Therefore, here we mainly outline the effects of MDMs on five IMIDs: inflammatory bowel disease (IBD), rheumatoid arthritis (RA), systemic lupus erythematosus (SLE), atopic dermatitis (AD), and asthma.

#### Inflammatory bowel disease

Inflammatory bowel disease (IBD), which mainly includes Crohn’s disease and ulcerative colitis, is a group of chronic inflammatory intestine disorders. Metabolomic analyses have revealed the alterations of MDMs level in IBD patients, contributing to IBD pathogenesis.^51,127–129^

SCFAs regulate intestinal inflammation by modulating T cell responses in various experimental colitis models, though their effects remain controversial. SCFAs can influence the size and function of colonic Treg populations, reducing the severity of T cell transfer model of colitis in mice.^62,65^ Interestingly, also in T cell transfer model of colitis in mice, butyrate leads to mild colitis by promoting Th1 cells while inhibiting Th17 cells.^71^ Further blockade of IL-10 signaling exacerbates colitis, underscoring IL-10‘s critical role in maintaining intestinal homeostasis.^71^ In dextran sulfate sodium salt (DSS)-induced acute mouse colitis, Kespohl et al. have reported that butyrate exacerbates colitis,^68^ while Sun et al. demonstrated that SCFAs like acetate or butyrate administered in drinking water can attenuate colitis.^21^ It should be emphasized that SCFAs receptors are instrumental in these effects, with receptor deficiencies leading to more severe colitis and even carcinogenesis.^17^ The diverse effects of SCFAs on colitis reflect a balance between their pro- and anti-inflammatory roles, influenced by the immune environment, SCFA types, concentrations, and receptor presence.

Dysregulation of tryptophan is also noted in IBD patients,^130^ with decreased fecal IAA^31^ and reduced serum IPA in active UC.^131^ Notably, indole derivatives like ILA, IPA, and IAA possess the ability to mitigate intestinal inflammation and modulate the gut microbiota in both DSS-induced and IL-10 spontaneous colitis models.^132^ These metabolites, acting *via* the AhR, promote anti-inflammatory cytokine IL-22 in colonic T cells and innate lymphoid cells (ILCs), reducing colitis.^31,91^ Moreover, ILA silences intestinal Th2 and Th17 inflammatory responses,^92^ further supporting the critical role of tryptophan derivatives in intestinal inflammation regulation.

Changes in microbiota mediated BA metabolism are also observed in IBD patients in some metabolomics analyses.^50,51,127^ In IBD patients, some secondary BAs as well as the bacterial genes required for their biosynthesis such as 3α-hydroxysteroid dehydrogenase gene are significantly reduced.^103,105^ The Th17/Treg balance is important in the modulation of BAs on intestinal homeostasis. Supplementation of certain BAs including isoalloLCA has been shown to ameliorate inflammatory colitis through upregulating Treg *via* BA receptors.^104,106^ Meanwhile, some BAs, such as 3-oxoLCA and isoLCA, can suppress Th17 cell differentiation to inhibit colitis development.^102–104^ The importance of BA receptor pathways in controlling colitis is highlighted by the severe colitis observed in *Vdr* ^flox/flox^*Foxp3*^YFP-cre^ mice.^106^

#### Rheumatoid arthritis and systemic lupus erythematosus

MDMs orchestrate autoimmune diseases. In rheumatoid arthritis (RA) development, butyrate reduces disease severity in a collagen-induced arthritis model by modulating the Th17/Treg balance^133^ and promotes T_FR_ cell differentiation in mice.^82^ Additionally, Jiang et al. have further revealed that sinomenine (SIN) can ameliorate mouse RA by regulating the gut microbiota – tryptophan metabolites – AhR axis, restoring Th17/Treg balance and improving intestinal barrier function.^90^ These findings underscore the importance of gut-joint interactions in RA pathogenesis, highlighting promising therapeutic avenues targeting key metabolites derived from gut microbiota. In systemic lupus erythematosus (SLE) mouse model, Josephine et al. have suggested that, tryptamine, a microbial metabolite of tryptophan, could activate autoreactive pathogenic CD4^+^ T cells, driving lupus progression.^98^

#### Atopic dermatitis

Atopic dermatitis (AD) is one of the most prevalent inflammatory skin diseases characterized by recurrent, pruritic, localized eczema, often with seasonal fluctuations.^134^ T cell responses, particularly Th2-driven inflammation, are central to its pathogenesis.^134^ A series of metagenomic and metabolomics studies indicate that microbiota and MDMs are also involved in the pathogenesis process and their levels vary in lesioned skin of patients with AD.^95,135^ Probiotics-derived propionates can promote differentiation of Foxp3^+^ Tregs to reduce Th1/Th17 type inflammation and ameliorates skin allergies.^16^ Tryptophan metabolites like IAld, produced by skin microbiota, suppress abnormal Th2 responses and alleviate dermatitis through AhR activation.^95^ This reveals that tryptophan metabolites derived from the skin microbiota play a regulatory role in skin inflammation. Similarly, gut-derived metabolites such as ILA, IPA, and I3C also suppress Th2-biased immune responses, alleviating AD symptoms, though these benefits are lost with AhR inhibition.^96,97^

#### Asthma

Propionate and butyrate suppress Th2 and Th9 cells, reducing allergic airway disease (AAD) severity in mice.^18,74^ Interestingly, acetate promotes Tregs to prevent the development of AAD with maternal high-fiber diets potentially influencing asthma susceptibility in offspring.^67^ Bacterial metabolites such as 12,13-diHOME may disrupt immune tolerance, contributing to childhood asthma.^119^

### Infection

Dietary factors have been closely linked to the anti-infection capabilities. Siracusa et al. find that a short-term shift from a regular diet to an energy-dense diet, high in animal fat and low in fiber, can reduce microbial SCFAs, leading to mucosal and systemic CD4^+^ T cell suppression and creating a window of vulnerability for pathogenic infections.^75^ However, reintroducing dietary fiber rewires T cell metabolism and restores mucosal and systemic CD4^+^ T cell functions.^75^ Moreover, supplementing dietary fiber to produce SCFAs can also enhance CD8^+^ T cell response during infections, such as influenza and herpes simplex virus type 1 (HSV-1) infection, helping resolve these infections more effectively.^20,78^ CLA can induce intraepithelial restored ileal CD4^+^CD8αα^+^ IELs to resist intestinal infections.^118^ Additionally, bacterial riboflavin (Vitamin B2) can be utilized by MAIT cells to detect microbial infections.^121^ These findings suggest that maintaining a diet rich in fiber can strengthen the immune system and improve the body’s ability to fight infections.

Overall, these studies reveal that the MDMs-T cells axis is involved in the development and treatment of cancer, IMIDs and infection mainly in a receptor-dependent manner. Hence, applications of MDMs or targeting MDMs receptors represent a potential avenue for combating various diseases. Due to space limitations, we have summarized the relationship between MDMs and T cells, as well as their roles in various diseases, in the **Supplementary Table**.

## Advances on research strategies for microbiota-dependent metabolites

### Detection and function characterization of microbiota-dependent metabolites

#### Microbiome-metabolomics

**Figure 3.**
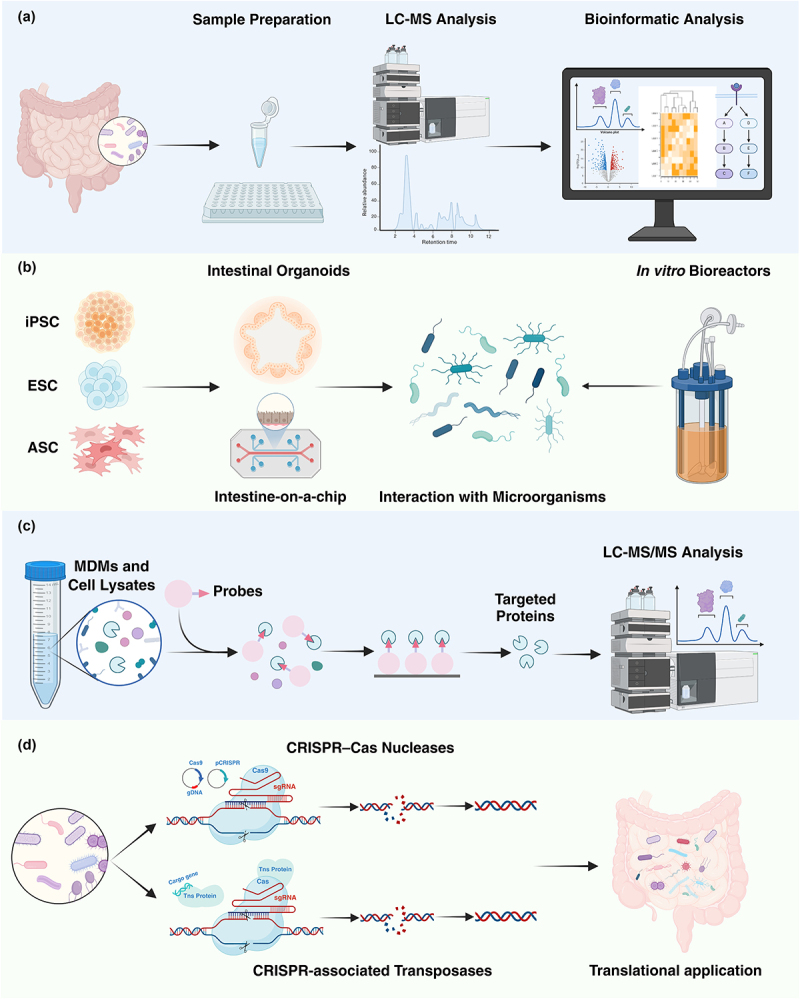
Advances on research strategies for microbiota-dependent metabolites. (a) Metabolomics analysis is a critical tool for discovering new metabolites and elucidating microbe–host interactions; (b) *in vitro* bioreactors, organoids and organ-on-a-chip provide controlled environments that simplify the complexity of microbiome analysis to investigate the host–microbe interactions. (c) Chemical proteomics is a powerful technique for the identification of small-molecule-interacting proteins and have gained popularity in elucidating targets of signaling metabolites. Microbiota-dependent metabolites – including tryptophan and BAs – have been converted to chemical probes for profiling their interacting proteins. (d) Gene manipulation is a vital method for single-gene interrogation within a complex microbiome and for regulating metabolite production at the genetic level, including CRISPR-Cas and transposase-based systems.

Abbreviations: ASC, adult stem cell; CRISPR, clustered regularly interspaced short palindromic repeats; ESC, embryonic stem cell; iPSC, induced pluripotent stem cell; LC-MS/MS, liquid chromatograph-mass spectrometer/mass spectrometry; MDMs, microbiota-dependent metabolites. (Created with bioRender.com)

Metabolomics analysis is a critical tool for discovering new metabolites and elucidating microbe–host interactions.^50,136^ Targeted metabolomics has been widely applied in analyses on known compounds. Untargeted metabolomics, in contrast, offers broader insights into novel MDMs, enabling a more comprehensive understanding of their interactions with the host.^137^ Mass spectrometry (MS)-based metabolomics is central to MDM characterization, with spectral libraries, substructure assignment, and advanced *in silico* tools used for metabolite annotation.^137^ Data from MS can then be analyzed for correlation or pathway prediction, linking metabolites to specific microorganisms (Figure 3a). For instance, Han et al. have developed an integrated MS-pipeline focusing on the microbiome, using machine learning to uncover a previously unknown type of metabolism in Bacteroides and identify candidate biochemical pathways through comparative genomics.^138^ High-throughput metabolomics techniques now allow for the characterization of over 800 MDMs, profoundly advancing our understanding of microbe–host interactions.^139^

#### Bioinformatic databases

Multiple databases provide MDM annotations with detailed information on human microorganisms, pathways, metabolite properties, and potential targets, including KEGG,^140^ MiMeDB,^141^ Natural Products Atlas,^142^ and LOTUS.^143^ However, these databases offer limited support for untargeted metabolomics research or fully characterizing certain metabolites. MicrobeMASST, a new search tool, allows users to search known and unknown MS/MS spectra and link them to their respective microbial producers *via* MS/MS fragmentation patterns,^144^ facilitating discovery without prior knowledge.

Metabolic pathway exploration is critical for studying MDM regulation. Several databases offer bioinformatic tools for visualizing, interpreting, and analyzing these pathways, including KEGG, HMDB, BioCyc, MetaCyc, Reactome, MetaboAnalyst, and SMPDB,^140,145–149^ which can guide subsequent experiments by suggesting possible metabolite-activated signaling pathways (Figure 3a).

#### In vitro *bioreactors and organ models*

The microbial environments are complex, which presents significant research challenges. The appearance of *in vitro* bioreactors and organ models^150,151^ offers controlled environments to reduce the complexity of microbiome analysis. However, these models often lack microbiome–host interactions. To address this, organoids and organ-on-a-chip have been developed to simulate more complex environments and host–microbe interactions.^152^ Advanced models now enable the recreation of complex physiological interactions through co-culture and multi-organ systems, such as the microfluidics-based HuMix model, which has shown that microbiome-derived SCFAs modulate CD4^+^ T cells.^153^ Although these *in vitro* models offer a simplified approach to studying gut microbiota–host interactions, their findings must be validated *in vivo*, such as with gnotobiotic animal models, which are widely used to study host-microbe molecular interactions^154^ (Figure 3b).

### Identifying the interaction mechanisms between microbiota-dependent metabolites and hosts

#### Chemical proteomics

MDMs are typically small molecules, making it essential to identify their targets to understand their regulatory effects. Traditional proteomics, which screens for potential targets by comparing protein expression changes after small molecule treatment, is not highly efficient. Chemical proteomics, which uses chemical probes that directly bind to target proteins, offers a more effective approach while minimizing nonspecific interactions. These probes typically include a reactive group (derived from the target molecule), a reporter tag, and a linker that sometimes is cleavable, to connect the reactive group and the reporter tag.^155^ Chemical probes come in various forms, including immobilized probes, activity-based probes, click chemistry probes, and photoaffinity probes, as well as non-labeling approaches.^155^ After probe synthesis and target enrichment, target proteins are identified using MS-based proteomics^156^ and protein microarrays.^157^ Chemical proteomics has proven effective in identifying targets in MDM research (Figure 3c). For instance, Fan et al. have designed photo-affinity probes using trans-vaccenic acid (TVA) as a parental molecule to bear a photo-reactive diazirine group and a tail alkynyl group to identify the TVA-binding protein target GPR43 on CD8^+^ T cell.^158^ In studying tryptophan metabolites, photoaffinity probes of IAA (x-alk-IAA) and TA (x-alk-TA) are applied to identify interacting proteins, including metabolic enzymes, transporters, immune sensors, and GPCRs.^159^

#### GPCR-based platforms

MDMs mostly act through GPCRs. The PRESTO-Tango platform, an open-source resource for parallel and simultaneous interrogation of the druggable GPCRome, has been proven valuable in screening human gut microbes for ligands that activate human GPCRs, facilitating the discovery of novel MDMs.^160,161^ PRESTO-Salsa, an enhanced version of PRESTO-Tango, employs a highly multiplexed screening method that links GPCR activation to the transcription of unique nucleic acid barcodes, enabling the simultaneous evaluation of hundreds of GPCRs in a single pool *via* next-generation sequencing.^162^ This technology dramatically increases screening throughput with reducing sample costs, enabling unprecedented exploration of GPCRome-wide bioactivities across diverse sample types.^162^

#### Stable isotope probing

MDMs interact with the host at various sites, affecting different tissues and organs. Stable isotope probing (SIP) is a powerful strategy that can trace active metabolic production and elucidate microorganism fates, even without prior knowledge of the MDMs.^163^ Combined with multi-omics, molecular techniques, or sequencing-based approaches, SIP enables researchers to explore metabolite sources, production capacities, and regulatory mechanisms, providing insights into how specific microbial metabolites regulate host homeostasis, disease and treatment outcomes.^164–167^

#### Genetic manipulation

Hundreds of microbiota genes are linked to host biology and diseases. Genetic manipulation (GM) is a vital method for single-gene interrogation and metabolite regulation at the genetic level. CRISPR-Cas and transposase-based systems are currently the preferred GM tools.^168,169^ GM of microorganisms offers several advantages, including clarifying metabolite sources, reducing interference with the host’s intestinal microenvironment and improving the safety of clinical applications.^168^ Additionally, *in situ* metabolite generation through GM better simulates normal production and dosage conditions, reducing the limitations of exogenous metabolites^168^ (Figure 3d). These benefits make GM of microorganisms crucial for studying gut microbiota–host interactions and for clinical applications. For instance, Jin et al. discover the role of *baiH* (a commensal gene for bile acid synthesis) in regulating colon inflammation by deleting it in a complex microbiome.^170^ Knocking out the gene responsible for the production of branched short-chain fatty acids in *C. sporogenes* shows that these microbial products have immunoglobulin A-modulatory activity.^171^ The latest research identified genes necessary for *F. nucleatum* adherence to CRC cells through a random mutagenesis library using the mariner-based transposon system.^172^

## Perspective

### Bridging prokaryotes and mammals: the role of MDMs

Microbiota-dependent metabolites (MDMs) represent a unique and groundbreaking class of biochemical compounds that are unlike any previously known metabolites produced solely by the human body. These metabolites are entirely new substances, generated or modified by the microbiota – particularly by prokaryotic bacteria residing within the human gut or other body sites – through their metabolic processing of various substrates, such as dietary components. What makes MDMs especially intriguing is their role as cross-kingdom regulatory signals that bridge the metabolic and physiological worlds of prokaryotes and eukaryotes. By acting as intermediaries between the microbial community and the host, MDMs serve as molecular messengers that regulate a wide range of biological processes in human, from immune modulation to metabolic pathways and even neurological functions. This cross-domain signaling is unprecedented, as MDMs enable bacterial communities to influence human biology in ways that were previously unimaginable, establishing a novel regulatory connection between bacteria and humans at a fundamental and biochemical level.

### Challenges in understanding MDMs: uncharacterized mechanisms

**Figure 4.**
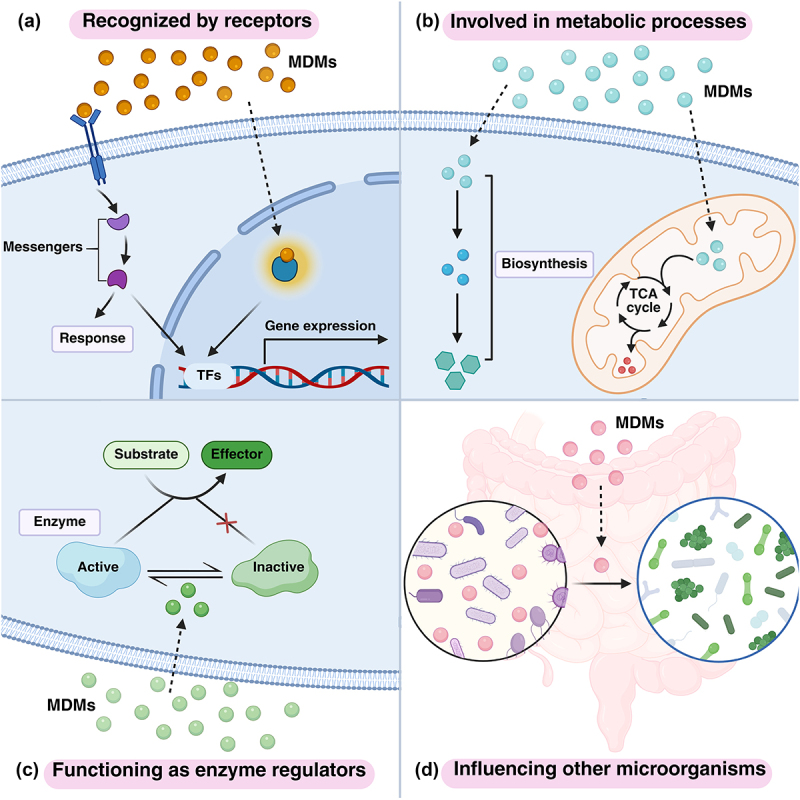
Current understanding for mechanisms of microbiota-dependent metabolites. Microbiota-dependent metabolites can exert their functions on T cell in 4 main ways: (a) serving as signaling molecules recognized by receptors; (b) acting as substrates or reactants in metabolic pathways such as biosynthesis; (c) functioning as enzyme regulators (either promoting or inhibiting enzymatic activity); (d) influencing the composition and function of other microbes.

Abbreviations: MDMs, microbiota-dependent metabolites; TCA, tricarboxylic acid cycle; TFs, transcription factors. (Created with bioRender.com)

Despite their importance, our understanding of MDMs remains limited. There are still many unidentified metabolites to be recognized. The functions of these metabolites, along with the mechanisms by which they are produced, are largely unknown. Furthermore, the way in which hosts – be they human, animal, or otherwise – perceive and respond to these metabolites is an area ripe for exploration. The current mechanisms of action of microbial metabolites can be categorized into several main types (Figure 4): 1) Serving as signaling molecules recognized by receptors; 2) Acting as substrates or reactants in metabolic processes such as biosynthesis; 3) Functioning as enzyme regulators (either promoting or inhibiting enzymatic activity); 4) Influencing the composition and function of other microbes. However, there are many gaps in knowledge regarding the underlying mechanisms, such as the receptors for metabolites, their binding modes, and their impact on the host’s overall metabolism. Utilizing high-throughput methods such as PRESTO-Tango and microbiome gene manipulation techniques can help better elucidate the production and action pathways of MDMs.

### MDMs and T cell in different microenvironments: location determines function

Just as the location of a property is vital when purchasing a house, the positioning and distribution of metabolites within the body are of critical importance in understanding their roles in health and diseases. The precise and accurate measurement of metabolite concentrations across different tissues, organs, and microenvironments presents a fundamental challenge in the future research. This challenge is compounded by the complexity of the body’s internal environment, where metabolites do not act in isolation but interact with a diverse array of cells, including T cells. T cells themselves are highly heterogeneous, with various subpopulations that perform distinct functions depending on the microenvironment in which they reside. The intricate interplay between these immune cells and microbial metabolites remains an area that is not fully understood. How these metabolites influence T cell behavior, function, and distribution across different microenvironments is a question that requires further exploration.

### Host–microbiome interactions: a chicken-or-egg dilemma

The age-old question, “Which came first, the chicken or the egg?”, finds a new context in the study of host–microbiome interactions. This question can be rephrased in this field as: Does the microbiome shape the host, or does the host’s physiology select for certain microbial communities? This is not merely a theoretical question; it has profound implications for our understanding of health and disease. To unravel this complex interplay, researchers must adopt a life cycle perspective, considering how these interactions evolve over time and across different stages of life. This will require robust epidemiological studies and carefully designed experiments that can provide the evidence needed to draw definitive conclusions. MDMs, as entirely new substances produced or modified by the microbiota, undoubtedly offer a fresh perspective for understanding the complex interactions between microbiota and their hosts. Research on MDMs provides solid causal evidence, reinforcing the significance of the microbiota and its derivatives. At the same time, it offers insights for studying how the host regulates the composition and structure of the microbiota.

### Microbial ecology and therapeutic potential of MDMs

Within the microecosystem, there exists a delicate balance of competition and cooperation among microbial strains. These interactions are crucial in determining how metabolites are produced and utilized. Understanding how different microbial strains collaborate – or compete – to produce metabolites is essential for developing strategies to manipulate the gut microecosystem effectively. This knowledge could pave the way for new therapies that target specific metabolic pathways, either by enhancing the production of beneficial metabolites or by reducing harmful ones. Our group reported that the metabolic cooperation between *Lactobacillus* and *Clostridium* can efficiently produce IPA and influence the epigenetic modifications of host T cells and immune checkpoint therapy for solid tumors. This provides a novel approach for combination probiotic therapies. The clinical translation of MDMs into therapeutic interventions is, therefore, a key area of research. It requires not only a deep understanding of microbial ecology and metabolism but also a systemic approach to manipulating these processes in a way that is both safe and effective for patients.

## Abbreviations

3-HAA: 3-hydroxyanthranilic acid
3 H-Kyn: 3-hydroxykynurenine
5-HT: Serotonin
AAD: Allergic airway disease
AD: Atopic dermatitis
AhR: Aryl hydrocarbon receptor
APC: Antigen-presenting cell
ArAT: Aromatic amino acid aminotransferase
BA: Bile acid
BSH: Bile salt hydrolase
CA: Cholate
CAR: Chimeric antigen receptor
CDCA: Chenodeoxycholate
CLA: Conjugated linoleic acid
CLNA: Conjugated linolenic acid
CNS: Conserved noncoding sequence
CRC: Colorectal cancer
CTL: Cytotoxic T lymphocyte
DC: Dendritic cell
DCA: Deoxycholic acid
DCA: Deoxycholic acid
DP IEL: Double-positive intraepithelial lymphocyte
DSS: Dextran sulfate sodium salt
FA: Fatty acid
T_FR_: Follicular regulatory T cell
T_FH_: Follicular helper T cell
FXR: Farnesoid X receptor
GLUT1: Glucose transporter 1
GPBAR1: G protein-coupled bile acid receptor 1
GPCR: G-protein-coupled receptor
GPR: G protein receptor
HDAC: Histone deacetylase
HIF-1α: Hypoxia inducible factor 1 alpha
HSDH: Hydroxysteroid dehydrogenase
HSV: Herpes simplex virus
I3C: Indole-3-carbaldehyde
IA: Indoleacrylic acid
IAA: Indole-3-acetic acid
IAld: Indolealdehyde
IBD: Inflammatory bowel disease
ICB: Immune checkpoint blockade
IEL: Intraepithelial lymphocyte
IFN-γ: Interferon-γ
IL: Interleukin
ILA: Indole-3-lactic acid
ILC: Innate lymphoid cell
IMID: Immune-mediated inflammatory disease
IPA: Indole-3-propionic acid
Kyn: Kynurenine
LA: Linoleic acid
LAI: Linoleate isomerase
LCA: Lithocholic acid
LCFA: Long-chain fatty acid
LNA: Linolenic acid
M1: Classical activated macrophages
MAIT: Mucosal-associated invariant T cell
MDM: Microbiota-dependent metabolite
MitoROS: Mitochondrial reactive oxygen species
MR1: MHC class I like related molecule
MS: Mass spectrometry
MS: Multiple sclerosis
NKT cell: Natural killer T cell
PDAC: Pancreatic ductal adenocarcinoma
PMCA: Plasma membrane Ca^2+^ ATPase
PXR: Pregnane X receptor
RA: Rheumatoid arthritis
RORγt: Retinoic acid receptor-related orphan nuclear receptors gamma t
SCFA: Short-chain fatty acid
SIN: Sinomenine
SLE: Systemic lupus erythematosus
TAM: Tumor-associated macrophage
TGF-β: Transforming growth factor-β
TGR: Takeda G-protein-coupled receptor
Th: T helper cell
TMA: Trimethylamine
TMAO: Trimethylamine N-oxide
TME: Tumor microenvironment
TNBC: Triple-negative breast cancer
Treg: Regulatory T cell
TVA: Trans-vaccenic acid
VDR: Vitamin D receptor

## Supplementary Material

Supplemental Material
