# Supplementary material for: Microbiota-dependent metabolites – New engine for T cell warriors
Source: Gut Microbes. 2025 Jun 30;17(1):2523815. doi: 10.1080/19490976.2025.2523815 (PMC12218545; doi:10.1080/19490976.2025.2523815)
Supplement: Supplemental Material [file KGMI_A_2523815_SM8840.docx]

| Table. Microbiota-Dependent Metabolites Regulated T Cell in Diseases | | | | | |
| --- | --- | --- | --- | --- | --- |
|  | **Disease** | **MDM** | **Regulated T Cell Type** | **Function** | **Ref.** |
| Cancer | Gastric Cancer | Butyrate | Antitumor cytotoxic CD8^+^ T cells /CAR T cells | Enhancing the killing function of CD8^+^ T cells or CAR CD8^+^ T cells against GC cells | [1] |
|  | Cancer | Butyrate | Antitumor cytotoxic CD8^+^ T cells | Enhancing antitumor CD8 T cell responses and improving chemotherapy efficacy | [2] |
|  | Cancer | Pentanoate, Butyrate | Antitumor cytotoxic CD8^+^ T cells /CAR T cells | Enhancing the killing function of CD8^+^ T cells or CAR CD8^+^ T cells | [3] |
|  | Cancer | SCFAs | Colonic Th17 | Increasing colonic Th17 cell frequency and their IL-17A and IL-17F expression prior to tumor formation and potentiate intestinal tumorigenesis | [4] |
|  | Cancer | ILA | Antitumor cytotoxic CD8^+^ T cells | Enhancing killing function of CD8^+^ T cells | [5] |
|  | Pancreatic Ductal Adenocarcinoma | ILA, IAA | CD8^+^ T cell | Driving an immunosuppressive phenotype in TAMs and suppressing CD8^+^ T cell accumulation in the TME to support cancer progression | [6] |
|  | Colorectal Cancer | ILA | Th17 | Suppressing Th17 differentiation to inhibit CRC development. | [7] |
|  |  | IPA | CD8^+^ T cell | Modulating CD8^+^ T cells stemness and progenitor exhausted CD8^+^ T cells (Tpex) and improving ICB responsiveness | [8] |
|  |  | DCA | CD8^+^ T cells | Suppressing CD8^+^ T cells function to promote tumor growth | [9] |
|  |  | DCA, LCA | Treg | Activate the β-catenin/CCL28 axis in tumor cells thus inducing intra-tumoral immunosuppressive CD25^+^FOXP3^+^ Treg cells | [10] |
|  | Liver Cancer | Secondary BAs | NKT cell | Reducing CXCL16 on liver sinusoidal endothelial cells thereby decreasing NKT cells accumulation to promote cancer progression | [11] |
|  | Triple-negative Breast cancer | TMAO | CD8^+^ T cell | Enhancing CD8^+^ T cell-mediated antitumor immunity by inducing tumor cell pyroptosis | [12] |
|  | Cancer | Inosine | Th1, CD8^+^ T cell | Enhancing ICB therapeutic efficacy by promoting IFN-γ-producing Th1/CD8^+^ effector T cells | [13] |
| IMIDs | IBD(Colitis) | SCFAs (Butyrate) | Treg | Enhancing colonic Treg production and function to alleviate colitis | [14, 15] |
|  |  | SCFAs (Butyrate) | IL-10-producing T cells (Th1 and Th17) | Promote IL-10 secretion to alleviate colitis | [16, 17] |
|  |  | Butyrate | Th1 | Enhancing the expression of T-bet and IFN-γ thus exacerbating colitis | [18] |
|  |  | ILA, IAA | Colonic CD4^+^ T cells | Enhancing the production of IL-22 to reduce colitis | [19, 20] |
|  |  | ILA | Th2 and Th17 | Silencing intestinal Th2 and Th17 inflammatory responses | [21] |
|  |  | BAs (3-oxoLCA, isoLCA) | Th17 | Inhibit Th17 differentiation to ameliorate colitis developments | [22-24] |
|  |  | BAs | Colonic Treg | Increasing colonic Treg cell counts and ameliorating host susceptibility to inflammatory colitis | [25] |
|  | RA | Butyrate | Th17/Treg | Decreasing Th17/Treg ratio and inducing IL-10 production to reduce RA severity | [26] |
|  |  | Butyrate | T_FR_ | Enhancing T_FR_ cells to suppress autoantibody production, eventually ameliorating RA | [27] |
|  |  | IA, IPA, IAA | Th17/Treg | Decreasing Th17/Treg ratio to reduce RA severity | [28] |
|  | SLE | Tryptamine | CD4^+^ T cells | Activating autoreactive pathogenic CD4+ T cells to drive lupus progression | [29] |
|  | Skin Allergy | Propionates | Treg | Enhancing Tregs differentiation to reduce Th1/Th17 type inflammation and ameliorating skin allergies | [30] |
|  | AD | IAId, ILA, IPA, I3C | Th2 | suppress abnormal Th2 responses to alleviate AD | [31-33] |
|  | Asthma | Propionate | Th2 | Impairing Th2 differentiation to alleviate AD | [34] |
|  |  | Butyrate | Th9 | Impairing Th9 cells differentiation to prevent lung inflammation | [35] |
|  |  | Acetate | Treg | suppression of AAD by enhancing T-regulatory cell numbers and function | [36] |
|  |  | 12,13-diHOME | Treg | Decrease Treg to increase the lung inflammation | [37] |
| Infection | Infection | SCFAs | CD4^+^ T cell | Maintaining mucosal and systemic CD4^+^ T cell functions and immunity to infection | [38] |
|  | Influenza-infection | SCFAs | CD8^+^ T cell | Boosting CD8 T cell function to prevent infection | [39] |
|  | HSV-1 infection | SCFAs | CD8^+^ T cell | Priming anti-viral CD8 T cell response during HSV-1 infection | [40] |
|  | Intestinal infection | CLA | CD4^+^ CD8αα^+^ IELs | Inducing CD4^+^ CD8αα^+^ IELs to prevent intestinal infection | [41] |
|  | Microbial infection detection | Bacterial riboflavin | MAIT cells | Activating MR1-resticted MAIT cells to detect microbial infections | [42] |

**Reference**

1. Yu, X., et al., *Gut microbiota modulate CD8(+) T cell immunity in gastric cancer through Butyrate/GPR109A/HOPX.* Gut Microbes, 2024. **16**(1): p. 2307542.

2. He, Y., et al., *Gut microbial metabolites facilitate anticancer therapy efficacy by modulating cytotoxic CD8(+) T cell immunity.* Cell Metab, 2021. **33**(5): p. 988-1000.e7.

3. Luu, M., et al., *Microbial short-chain fatty acids modulate CD8(+) T cell responses and improve adoptive immunotherapy for cancer.* Nat Commun, 2021. **12**(1): p. 4077.

4. Brennan, C.A., et al., *Fusobacterium nucleatum drives a pro-inflammatory intestinal microenvironment through metabolite receptor-dependent modulation of IL-17 expression.* Gut Microbes, 2021. **13**(1): p. 1987780.

5. Zhang, Q., et al., *Lactobacillus plantarum-derived indole-3-lactic acid ameliorates colorectal tumorigenesis via epigenetic regulation of CD8(+) T cell immunity.* Cell Metab, 2023. **35**(6): p. 943-960.e9.

6. Hezaveh, K., et al., *Tryptophan-derived microbial metabolites activate the aryl hydrocarbon receptor in tumor-associated macrophages to suppress anti-tumor immunity.* Immunity, 2022. **55**(2): p. 324-340.e8.

7. Han, J.X., et al., *Microbiota-derived tryptophan catabolites mediate the chemopreventive effects of statins on colorectal cancer.* Nat Microbiol, 2023. **8**(5): p. 919-933.

8. Jia, D., et al., *Microbial metabolite enhances immunotherapy efficacy by modulating T cell stemness in pan-cancer.* Cell, 2024. **187**(7): p. 1651-1665.e21.

9. Cong, J., et al., *Bile acids modified by the intestinal microbiota promote colorectal cancer growth by suppressing CD8(+) T cell effector functions.* Immunity, 2024. **57**(4): p. 876-889.e11.

10. Sun, L., et al., *Bile salt hydrolase in non-enterotoxigenic Bacteroides potentiates colorectal cancer.* Nat Commun, 2023. **14**(1): p. 755.

11. Ma, C., et al., *Gut microbiome-mediated bile acid metabolism regulates liver cancer via NKT cells.* Science, 2018. **360**(6391).

12. Wang, H., et al., *The microbial metabolite trimethylamine N-oxide promotes antitumor immunity in triple-negative breast cancer.* Cell Metab, 2022. **34**(4): p. 581-594.e8.

13. Mager, L.F., et al., *Microbiome-derived inosine modulates response to checkpoint inhibitor immunotherapy.* 2020. **369**(6510): p. 1481-1489.

14. Furusawa, Y., et al., *Commensal microbe-derived butyrate induces the differentiation of colonic regulatory T cells.* Nature, 2013. **504**(7480): p. 446-450.

15. Smith, P.M., et al., *The microbial metabolites, short-chain fatty acids, regulate colonic Treg cell homeostasis.* Science, 2013. **341**(6145): p. 569-73.

16. Chen, L., et al., *Microbiota Metabolite Butyrate Differentially Regulates Th1 and Th17 Cells' Differentiation and Function in Induction of Colitis.* Inflamm Bowel Dis, 2019. **25**(9): p. 1450-1461.

17. Sun, M., et al., *Microbiota-derived short-chain fatty acids promote Th1 cell IL-10 production to maintain intestinal homeostasis.* Nat Commun, 2018. **9**(1): p. 3555.

18. Kespohl, M., et al., *The Microbial Metabolite Butyrate Induces Expression of Th1-Associated Factors in CD4(+) T Cells.* Front Immunol, 2017. **8**: p. 1036.

19. Lamas, B., et al., *CARD9 impacts colitis by altering gut microbiota metabolism of tryptophan into aryl hydrocarbon receptor ligands.* Nature Medicine, 2016. **22**(6): p. 598-605.

20. Laursen, M.F., et al., *Bifidobacterium species associated with breastfeeding produce aromatic lactic acids in the infant gut.* Nat Microbiol, 2021. **6**(11): p. 1367-1382.

21. Henrick, B.M., et al., *Bifidobacteria-mediated immune system imprinting early in life.* Cell, 2021. **184**(15): p. 3884-3898.e11.

22. Yan, Y., et al., *Bacteroides uniformis-induced perturbations in colonic microbiota and bile acid levels inhibit TH17 differentiation and ameliorate colitis developments.* NPJ Biofilms Microbiomes, 2023. **9**(1): p. 56.

23. Paik, D., et al., *Human gut bacteria produce Τ(Η)17-modulating bile acid metabolites.* Nature, 2022. **603**(7903): p. 907-912.

24. Hang, S., et al., *Bile acid metabolites control T(H)17 and T(reg) cell differentiation.* Nature, 2019. **576**(7785): p. 143-148.

25. Song, X., et al., *Microbial bile acid metabolites modulate gut RORγ(+) regulatory T cell homeostasis.* Nature, 2020. **577**(7790): p. 410-415.

26. Kim, D.S., et al., *Attenuation of Rheumatoid Inflammation by Sodium Butyrate Through Reciprocal Targeting of HDAC2 in Osteoclasts and HDAC8 in T Cells.* Front Immunol, 2018. **9**: p. 1525.

27. Takahashi, D., et al., *Microbiota-derived butyrate limits the autoimmune response by promoting the differentiation of follicular regulatory T cells.* EBioMedicine, 2020. **58**: p. 102913.

28. Jiang, Z.M., et al., *Sinomenine ameliorates rheumatoid arthritis by modulating tryptophan metabolism and activating aryl hydrocarbon receptor via gut microbiota regulation.* Sci Bull (Beijing), 2023. **68**(14): p. 1540-1555.

29. Brown, J., et al., *Microbiota-mediated skewing of tryptophan catabolism modulates CD4(+) T cells in lupus-prone mice.* iScience, 2022. **25**(5): p. 104241.

30. Kang, H.J., et al., *Probiotics-derived metabolite ameliorates skin allergy by promoting differentiation of FOXP3(+) regulatory T cells.* J Allergy Clin Immunol, 2021. **147**(4): p. 1517-1521.

31. Yu, J., et al., *A tryptophan metabolite of the skin microbiota attenuates inflammation in patients with atopic dermatitis through the aryl hydrocarbon receptor.* J Allergy Clin Immunol, 2019. **143**(6): p. 2108-2119.e12.

32. Fang, Z., et al., *Limosilactobacillus reuteri Attenuates Atopic Dermatitis via Changes in Gut Bacteria and Indole Derivatives from Tryptophan Metabolism.* Int J Mol Sci, 2022. **23**(14).

33. Fang, Z., et al., *Bifidobacterium longum mediated tryptophan metabolism to improve atopic dermatitis via the gut-skin axis.* Gut Microbes, 2022. **14**(1): p. 2044723.

34. Trompette, A., et al., *Gut microbiota metabolism of dietary fiber influences allergic airway disease and hematopoiesis.* Nature Medicine, 2014. **20**(2): p. 159-166.

35. Vieira, R.S., et al., *Butyrate Attenuates Lung Inflammation by Negatively Modulating Th9 Cells.* Front Immunol, 2019. **10**: p. 67.

36. Thorburn, A.N., et al., *Evidence that asthma is a developmental origin disease influenced by maternal diet and bacterial metabolites.* Nat Commun, 2015. **6**: p. 7320.

37. Levan, S.R., et al., *Elevated faecal 12,13-diHOME concentration in neonates at high risk for asthma is produced by gut bacteria and impedes immune tolerance.* Nat Microbiol, 2019. **4**(11): p. 1851-1861.

38. Siracusa, F., et al., *Short-term dietary changes can result in mucosal and systemic immune depression.* Nat Immunol, 2023. **24**(9): p. 1473-1486.

39. Trompette, A., et al., *Dietary Fiber Confers Protection against Flu by Shaping Ly6c(-) Patrolling Monocyte Hematopoiesis and CD8(+) T Cell Metabolism.* Immunity, 2018. **48**(5): p. 992-1005.e8.

40. Lee, A.R., et al., *GPR41 and GPR43 regulate CD8(+) T cell priming during herpes simplex virus type 1 infection.* Front Immunol, 2024. **15**: p. 1332588.

41. Song, X., et al., *Gut microbial fatty acid isomerization modulates intraepithelial T cells.* Nature, 2023. **619**(7971): p. 837-843.

42. Kjer-Nielsen, L., et al., *MR1 presents microbial vitamin B metabolites to MAIT cells.* Nature, 2012. **491**(7426): p. 717-23.
